# The *Plasmodium* Lactate/H^+^ Transporter PfFNT Is Essential and Druggable *In Vivo*

**DOI:** 10.1128/aac.00356-23

**Published:** 2023-07-10

**Authors:** Heledd Davies, Bärbel Bergmann, Philipp Walloch, Cornelius Nerlich, Christian Hansen, Sergio Wittlin, Tobias Spielmann, Moritz Treeck, Eric Beitz

**Affiliations:** a Signalling in Apicomplexan Parasites Laboratory, The Francis Crick Institute, London, United Kingdom; b Bernhard-Nocht-Institute for Tropical Medicine, Hamburg, Germany; c Department of Pharmaceutical and Medicinal Chemistry, Christian-Albrechts-University of Kiel, Kiel, Germany; d Swiss Tropical and Public Health Institute, Allschwil, Switzerland; e University of Basel, Basel, Switzerland; f Instituto Gulbenkian de Ciência, Oeiras, Portugal

**Keywords:** *Plasmodium*, malaria, formate-nitrite transporter, lactate, proton, resistance, antimalarials, antimalarial agents

## Abstract

Malaria parasites in the blood stage express a single transmembrane transport protein for the release of the glycolytic end product l-lactate/H^+^ from the cell. This transporter is a member of the strictly microbial formate-nitrite transporter (FNT) family and a novel putative drug target. Small, drug-like FNT inhibitors potently block lactate transport and kill Plasmodium falciparum parasites in culture. The protein structure of Plasmodium falciparum FNT (PfFNT) in complex with the inhibitor has been resolved and confirms its previously predicted binding site and its mode of action as a substrate analog. Here, we investigated the mutational plasticity and essentiality of the PfFNT target on a genetic level, and established its *in vivo* druggability using mouse malaria models. We found that, besides a previously identified PfFNT G107S resistance mutation, selection of parasites at 3 × IC_50_ (50% inhibitory concentration) gave rise to two new point mutations affecting inhibitor binding: G21E and V196L. Conditional knockout and mutation of the PfFNT gene showed essentiality in the blood stage, whereas no phenotypic defects in sexual development were observed. PfFNT inhibitors mainly targeted the trophozoite stage and exhibited high potency in P. berghei- and P. falciparum-infected mice. Their *in vivo* activity profiles were comparable to that of artesunate, demonstrating strong potential for the further development of PfFNT inhibitors as novel antimalarials.

## INTRODUCTION

The growth of blood-stage malaria parasites depends on the unhindered release of lactate and its accompanying proton from the cell (1). Lactate is the sole end product of the parasite’s glycolytic energy metabolism (2). Until recently, the protein responsible for the export of lactate was unknown. We and others identified a member of the strictly microbial formate-nitrite transporter (FNT) family as the missing lactate/H^+^ transporter of *Plasmodium* spp. (3, 4). FNTs are channel-like, low-affinity, secondary-active transporters of weak acids which use a transmembrane proton gradient as a driving force for transport (5, 6). Each protomer of the homopentameric FNT complex (7) forms an individual transport unit that funnels lactate anions into vestibules toward positively charged lysine residues (5). The increasingly lipophilic environment inside the vestibule renders the substrate less acidic, enabling a proton to hop on for neutralization (8). The neutral acid molecule can then pass the rigid tube-like lipophilic core of the transport path of the protein (9). Lactate transporters of the human host, monocarboxylate transporters (MCT), are unrelated to the FNTs in terms of their sequence, structure, and substrate binding sites, and act by an alternating-access transport mechanism (10, 11).

Apicomplexan FNTs can be targeted and blocked by specific small molecules (12–14). The inhibitor class is based on the Malaria Box compound MMV007839 (Fig. S1 in the supplemental material) and exhibits nanomolar affinity on the FNTs from P. falciparum (PfFNT) (15) and the four other human-pathogenic *Plasmodium* spp. P. vivax, P. malariae, P. ovale, and P. knowlesi, as well as the rodent malaria parasite P. berghei (16). Treatment of P. falciparum 3D7 cultures with PfFNT inhibitors efficiently kills the parasites due to cessation of the energy metabolism and acidification of the cytosol (13).

We obtained preliminary evidence that PfFNT is the primary target of MMV007839 by force-selection of resistance with 3 × IC_50_ (50% inhibitory concentration) inhibitor dosing (12). Sequencing of the *fnt* gene isolated from the obtained resistant parasites revealed a point mutation that leads to the exchange of Gly107 with Ser. We hypothesized that the larger Ser residue collides with the phenol-hydroxyl moiety of MMV007839, and we were able to circumvent the resistance mutation by replacing the phenol ring with benzene (compound BH296) or pyridine (BH267.meta; Fig. S1) (17). The nitrogen atom of the pyridine ring accepts a hydrogen bond from the Ser-hydroxyl of the PfFNT G107S resistance mutant, regaining inhibition of the yeast-expressed protein in the nanomolar range. Initial P. falciparum 3D7 selection cultures treated with 3 × IC_50_ doses of BH267.meta were free of resistant parasites over an observation period of 30 days.

Although the molecular structure-function relationships of the FNT protein family and the binding modes of the inhibitors are well-characterized (18), data on the essentiality and druggability of PfFNT in an *in vivo* context were missing.

In this study, we extended the search for inhibitor-induced mutations of PfFNT, determined the developmental stage at which the inhibitors targeted the parasite, generated conditional PfFNT knockouts and conditional point mutations, and tested the *in vivo* efficacy of BH267.meta in mouse malaria models. We demonstrate essentiality of PfFNT in the trophozoite state and >99% activity of BH267.meta in mice, supporting further development of PfFNT inhibitors as antimalarials with a novel mode of action.

## RESULTS

### Additional mutations conferring resistance to PfFNT inhibitors.

**FIG 1:**
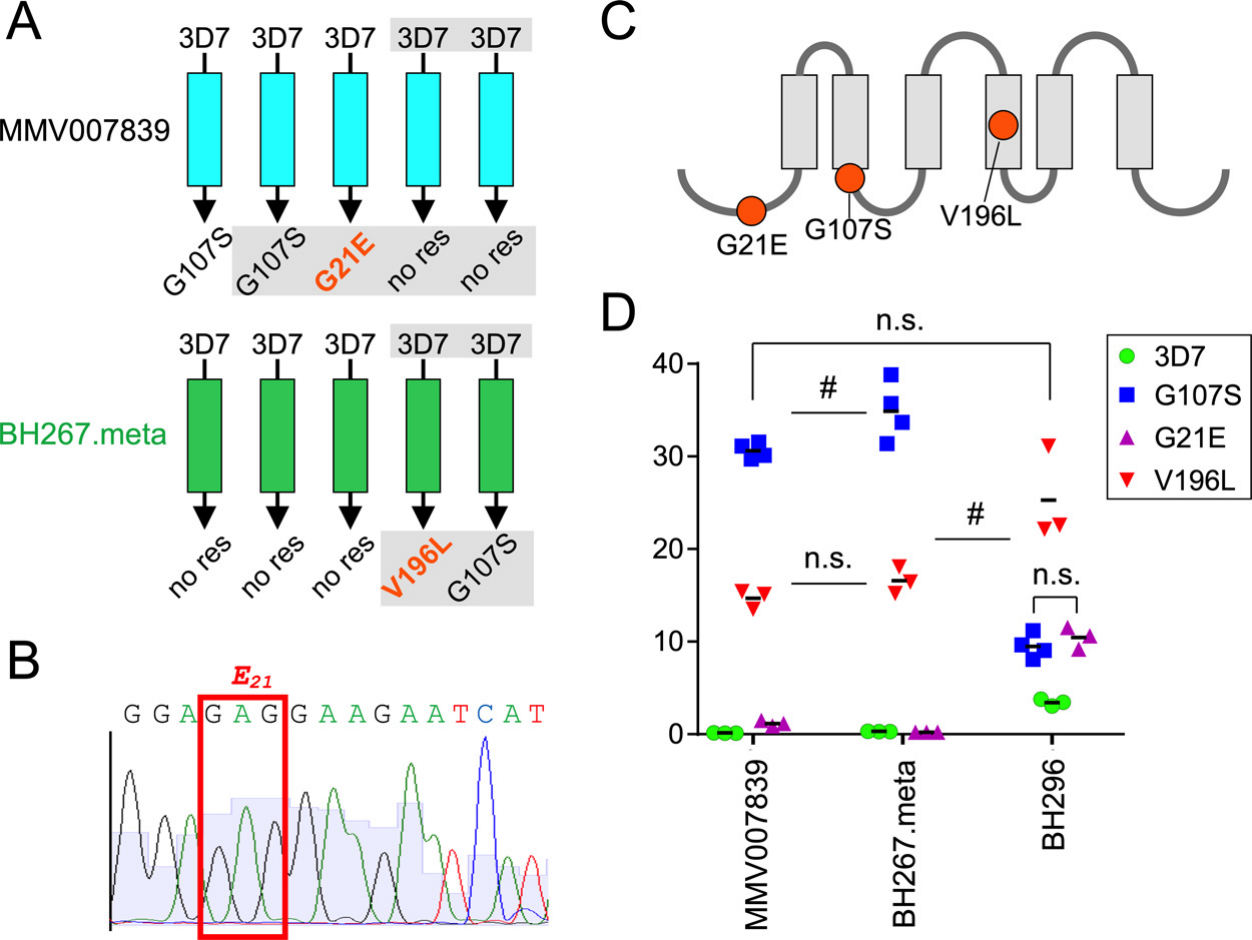
Novel mutations in PfFNT decrease drug sensitivity of the parasite. (A) Summary of attempts to raise resistant parasites using either MMV007839 or BH267.meta, including previous attempts (12, 17). Gray shaded areas show analyses done for this work; mutations not previously observed are in orange. (B) Sequencing electropherogram around the glutamate-21 GAG codon indicating that the majority of the surfaced parasites carry the G-to-A mutation at the second position. (C) Schematic showing the position of mutations in PfFNT in parasites with reduced sensitivity to the inhibitors tested. (D) EC_50_ values of the indicated drugs with the parasites of the indicated mutations. Each dot represents the EC_50_ calculated from one independently generated dose response curve. Non-significance (n.s.) and borderline significance (#) is indicated; see Table S1 in the supplemental materials for *P* values of all drug versus strain combinations.

In previous work, we were unable to obtain parasites resistant to BH267.meta (17). Two further attempts to select resistant parasites by exposing 3D7 wild-type parasites to 3 × IC_50_ of BH267.meta resulted in parasites surfacing under the drug (Fig. 1A). Sequencing of the *fnt* gene in these parasites revealed that one of the two lines had acquired a mutation resulting in the resistance-conferring G107S change that we and others had previously obtained from similar experiments using the original PfFNT inhibitor MMV007839 (12, 13). The other parasite line had a new mutation resulting in a V196L change in PfFNT. We also sequenced the *fnt* gene in two resistant lines that we had previously generated using 3 × IC_50_ MMV007839 (17). One of these parasite lines again had the G107S mutation, but the other had a different mutation which resulted in a G21E amino acid change in PfFNT (Fig. 1A). The sequencing electropherograms showed that without cloning, the majority of the parasites that surfaced after drug exposure carried the indicated mutation (example: the adenine position in the glutamate-21 GAG codon in Fig. 1B). In total, three PfFNT mutations appeared under drug pressure: G107S and V196L at the inhibitor binding site, and G21E, which lies outside the transport path at the N terminus of PfFNT (Fig. 1C) as indicated by the PfFNT structure (18).

Next, we assessed the EC_50_ (50% effective concentration) for MMV007839, BH296, and BH267.meta against parasites harboring the new PfFNT mutations and compared them to wild-type (3D7) parasites and parasites with the previously described PfFNT G107S mutation (12). While the parasites with the new V196L mutation showed a strongly reduced sensitivity to MMV007839 and BH267.meta, this loss of effectivity was only about half of that seen with the previously observed G107S mutation (Fig. 1D). In contrast, BH296 sensitivity of the parasites harboring the new V196L mutation was even lower than that of the parasites with the G107S mutation (Fig. 1D). The parasites with the G21E amino acid change had a more modest reduction in sensitivity to MMV007839 (8.4-fold) and BH296 (3.0-fold) compared to the 3D7 parasites and no alteration in the response to BH267.meta. Overall, BH296 was the least affected by the G107S and V196L mutations (2.8- and 7.4-fold increased IC_50_, respectively).

**FIG 2:**
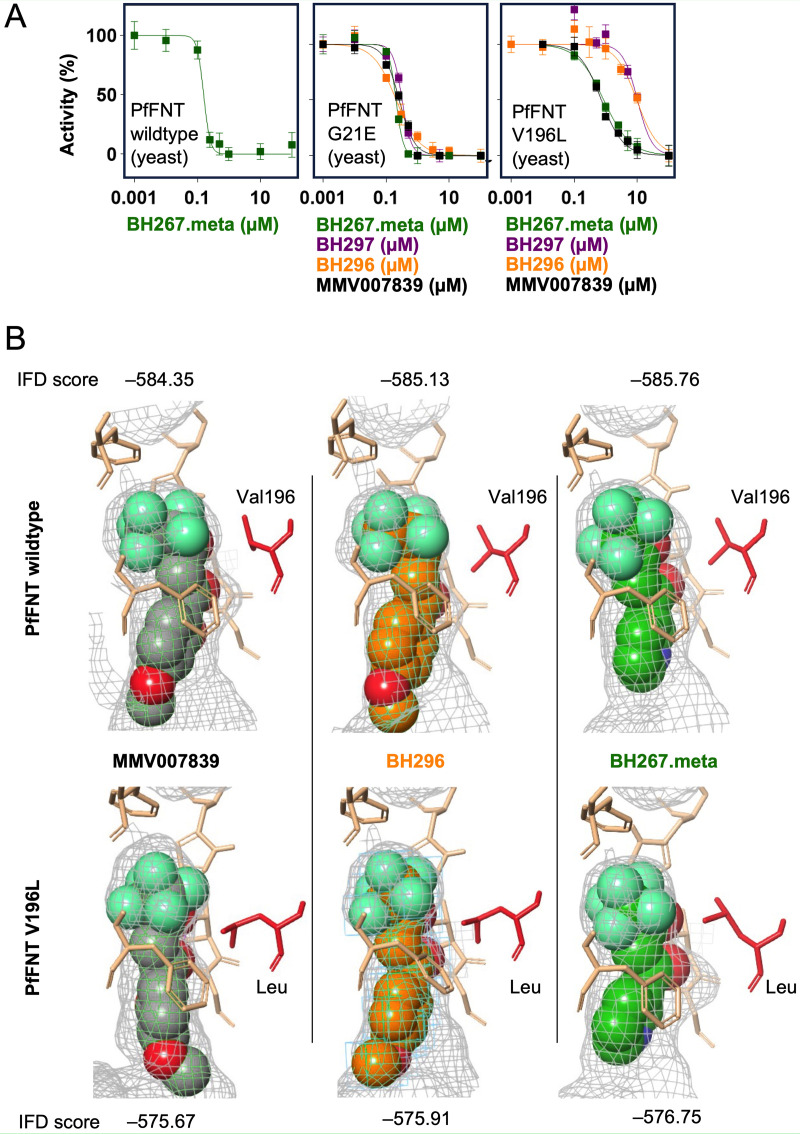
Activity of PfFNT inhibitors on mutated PfFNT expressed in yeast. (A) PfFNT wild-type and the variants G21E and V196L were expressed in yeast, and compound activity was determined by uptake inhibition of ^14^C-labeled lactate. *n* = 3, error bars denote standard error of the mean (SEM). (B) Virtual docking of PfFNT wild-type (PDB no. 7E27) and a generated PfFNT V196L model with MMV007839, BH296, and BH267.meta. Schrödinger-induced fit docking scores (IFD) are indicated for the highest-ranked docking pose for each protein-inhibitor pair.

We then expressed wild-type and mutant PfFNT in Saccharomyces cerevisiae yeast lacking endogenous monocarboxylate transporters for functional assays (Fig. S2A). We grew the transformed yeast in liquid medium with lactate as the sole carbon source (16). Despite some delay in the growth curve, the G21E mutation enabled lactate uptake at a similar level as PfFNT wild-type or the previously described G107S mutant, as seen by the achieved maximal cell density (plateaus in Fig. S2B). Expression of PfFNT V196L, however, led to a larger delay in the growth curve and a one-third decrease in the maximal cell density, indicating that this mutation impedes lactate transport. We also used yeast transformed with the newly detected PfFNT variants G21E and V196L to determine the activity of MMV007839, BH297, BH296, and BH267.meta on the inhibition of ^14^C-lactate transport (Fig. 2A). The data generally confirmed the findings of the *in vitro* IC_50_ determinations. PfFNT G21E was inhibited by all compounds (Fig. 2A, center) with similar IC_50_ values, in the range of 0.2 to 0.3 μM, compared to the PfFNT wild-type (Fig. 2A, left) (17). In contrast, the V196L mutation decreased the activity throughout (Fig. 2A, right). Here, BH297 was affected most strongly, with the IC_50_ shifting to 10.2 ± 1.9 μM, whereas the IC_50_ of MMV007839 and BH267.meta remained in the sub-micromolar range (0.73 ± 0.07 and 0.85 ± 0.05 μM, respectively).

We docked MMV007839, BH296, and BH267.meta into the PfFNT wild-type structure (18) and into a generated model of the V196L variant (Fig. 2B). Figure 2B shows the best ranked binding pose for each inhibitor after induced fit docking (IFD). The additional methylene unit of the leucine residue limits the available space at the binding site and requires some movement of the sidechain to accommodate the inhibitor. Two different conformations of the leucine sidechain were observed (Fig. 2B, left and center panel versus right panel). Overall, the binding poses were highly similar throughout, but the obtained docking scores were lower with PfFNT V196L than with the wild-type protein.

### PfFNT inhibitors attack the trophozoite stage.

**FIG 3:**
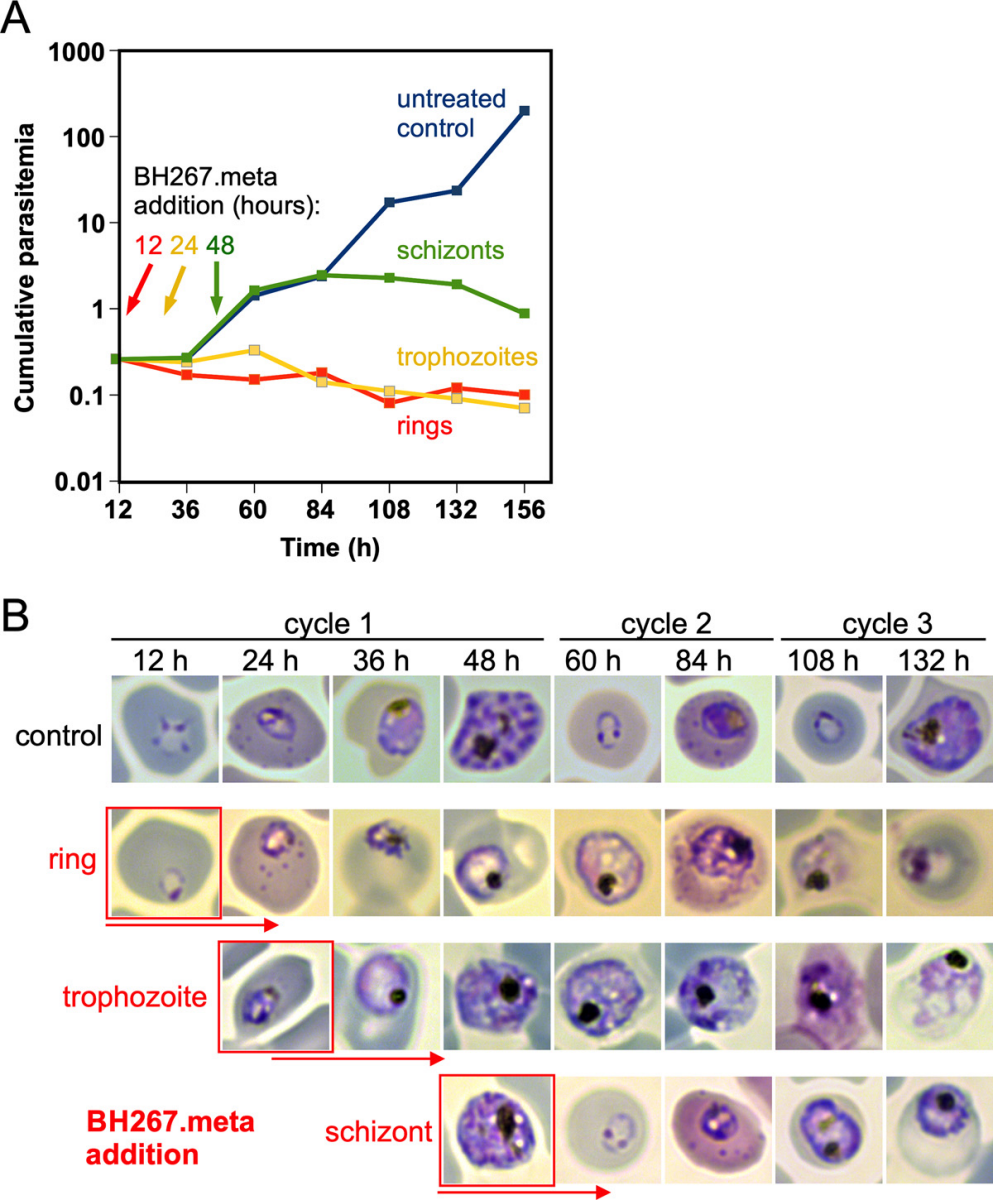
Determination of the parasite blood stage targeted by PfFNT inhibitors. (A) Growth of 3D7 parasites (cumulative parasitemia) as determined by flow cytometry of the same culture split into 4 parts, of which one was left untreated (control, blue) and one was treated with the IC_50_ of BH267.meta from the ring stage onward (red), one from the trophozoite stage onwards (yellow), and one once parasites had developed to the schizont stage (green). The starting point of drug treatment is indicated on the curves for each condition. Time after start of the experiment is shown in hours (h). (B) Example images from Giemsa smears taken at indicated time points. The stage of BH267.meta addition is shown in red boxes. Note that for the first development cycle, Giemsa smears were taken twice a day, whereas parasitemia (B) was measured only once per day but continued to 160 h after the start of the experiment. One of two similar experiments (see Fig. S2B and C).

To assess which blood stage is affected by PfFNT inhibitors, we synchronized parasites, subjected them to IC_90_ of BH267.meta at different stages, and tracked parasite proliferation (Fig. 3A). When BH267.meta was added to rings or trophozoites, the parasitemia did not increase while the control multiplied (Fig. 3A; Fig. S3). Inspection of the parasites at different time points in these samples indicated that the BH267.meta-exposed rings progressed to the trophozoite stage but did not produce viable schizonts (as evident from aberrant parasite morphology in Giemsa smears and a failure to produce novel rings) (Fig. 3B; Fig. S3). The exposed trophozoites also did not produce viable schizonts (Fig. 3B). The schizonts subjected to BH267.meta gave rise to new rings which developed into trophozoites but again failed to develop into viable schizonts (Fig. 3B; Fig. S3). Overall, this indicates that BH267.meta specifically affects the trophozoite stage of the parasite.

### PfFNT is essential for asexual growth but appears dispensable for the development of gametocytes.

**FIG 4:**
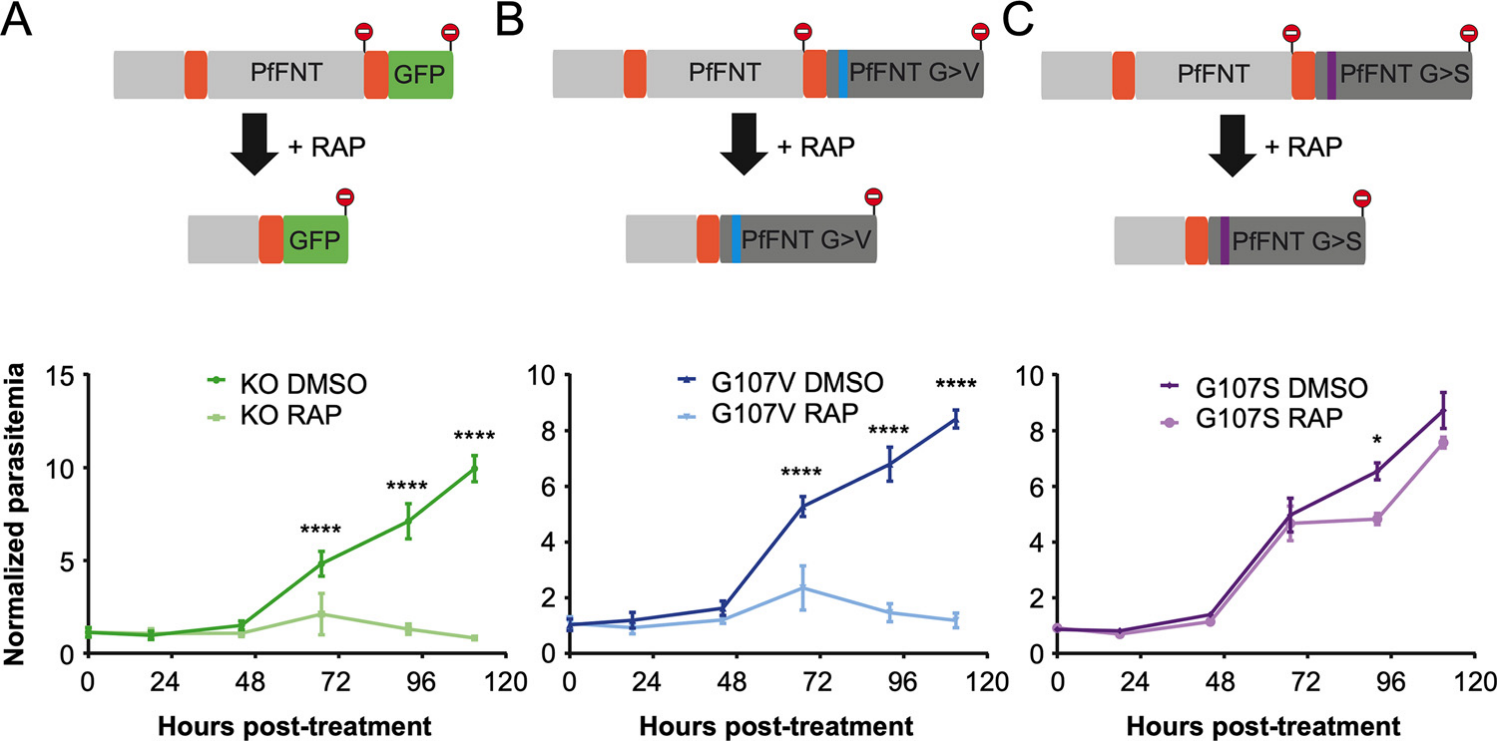
Generation and growth of conditional PfFNT knockout and mutation parasite lines. In a NF54 diCre line, the four C-terminal transmembrane domains of PfFNT were flanked with *loxP* introns. (A to C) Addition of rapamycin leads to the excision and insertion of GFP (A) (green) or a re-codonized version of the sequence carrying a G107V (B) (blue) or G107S mutation (C) (magenta). Lower panels show growth curves of the parasites expressing wild-type PfFNT (DMSO), and after conditional mutation of PfFNT by rapamycin treatment (RAP). Assays were performed on 3 clones (*n* = 3), and the mean values ± SEM are shown. Statistical significance was determined using paired one-way analysis of variance (ANOVA) of DMSO versus RAP parasitemia at each time point, followed by Sidak’s multiple-comparison post-test.

**FIG 5:**
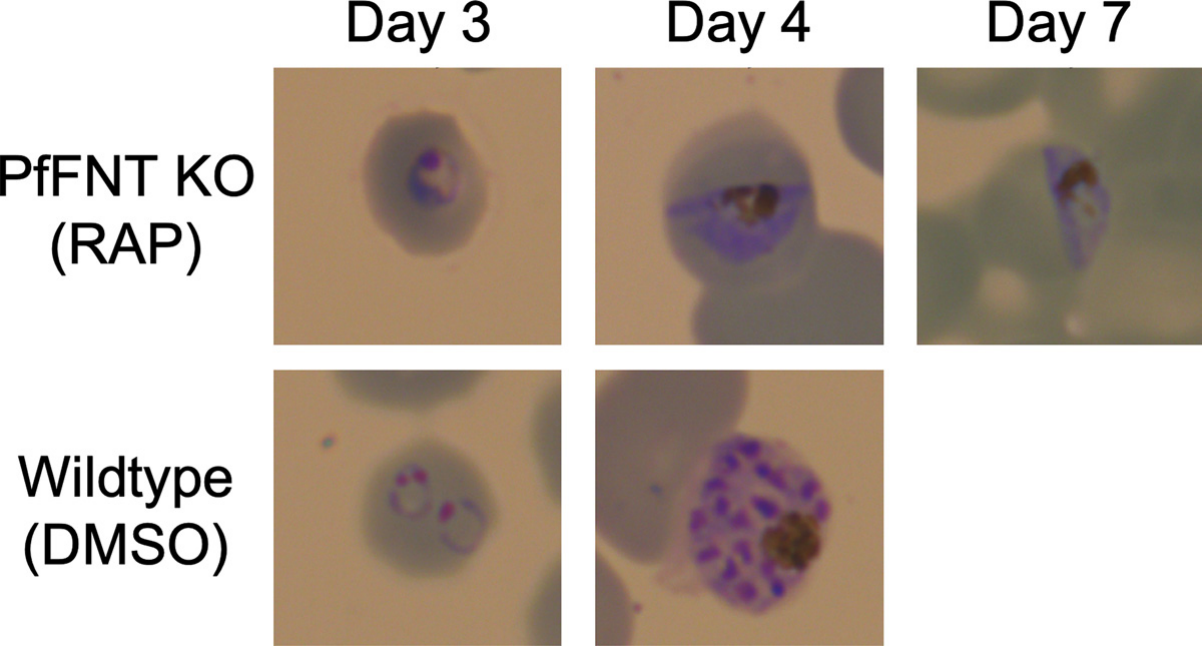
Development of parasites after conditional PfFNT knockout. Giemsa staining of wild-type parasites (lower panel, DMSO) shows normal development from rings to schizonts after 4 d (two cycles). Parasites with conditional PfFNT knockout after rapamycin treatment (upper panel, RAP) stall at the trophozoite stage of cycle 1 and fail to progress to the next cycle, but gametocytes appear to develop normally.

To verify the essentiality of PfFNT, we generated a conditional knockout line (Fig. 4A). In an NF54 diCre line, we flanked the four C-terminal transmembrane domains of PfFNT with *loxP* introns, allowing the conditional excision of this domain upon the addition of rapamycin (19). A green fluorescent protein (GFP) cassette comes into frame with the remaining N terminus of the gene upon excision, although in practice no GFP signal was observed, likely due to the instability of the remaining protein. Upon treatment with rapamycin in the ring stages, the majority of parasites did not progress to the next cycle and within two cycles no asexual stages remained, demonstrating the essentiality of PfFNT and lactate export for parasite asexual development (Fig. 4A and 5). Surprisingly, gametocytes continued to develop upon PfFNT deletion, appearing normal even at 7 days after rapamycin treatment, suggesting that lactate export is not essential for the sexual stages of development until this time point.

### Conditional mutation of PfFNT.

To establish whether mutation of PfFNT is the sole driver of resistance to the inhibitor compounds, we generated conditional mutants with amino acid changes to Gly107 (Fig. 4B and C). In this case, when rapamycin is added to the parasites, the four C-terminal transmembrane domains are excised, bringing a re-codonized version of the same sequence into frame to complement the deleted region. Into this second re-codonized sequence, we inserted one of two mutations, either G107S or G107V. While G107V was shown to inactivate the transporter in yeast, G107S is active yet confers resistance to PfFNT inhibitors (12).

As predicted, treatment of the G107V conditional mutant line with rapamycin phenocopied the gene knockout, with no asexual parasites surviving for more than two cycles (Fig. 4B). The G107S mutation did not appear to substantially impair parasite development; however, there was a reduction in growth during the second cycle, perhaps suggesting a small growth delay (Fig. 4C).

### Drug susceptibility of conditional PfFNT G107S mutants.

**FIG 6:**
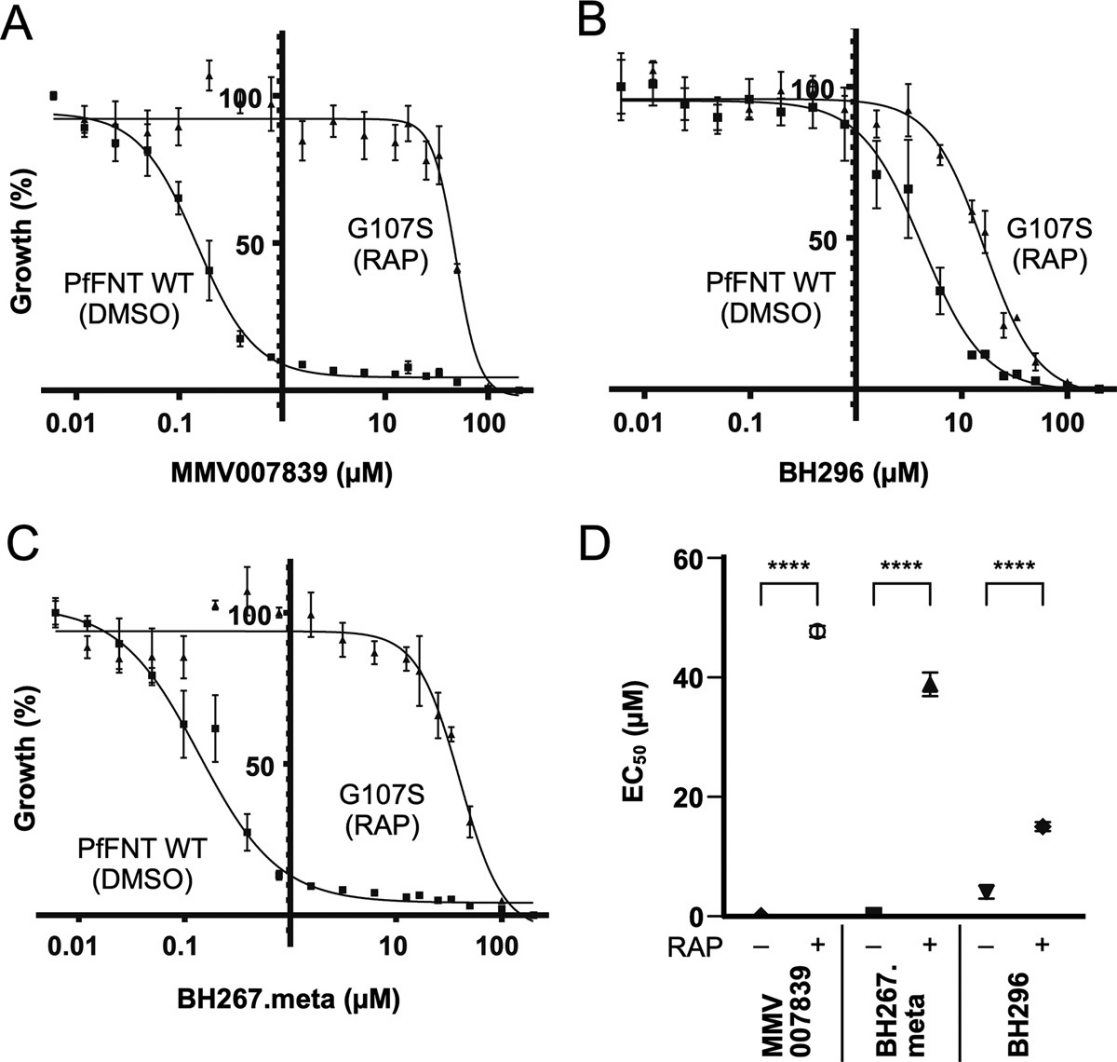
(A to C) Susceptibility against MMV007839 (A), BH296 (B), and BH267.meta (C) of parasites expressing conditional PfFNT G107S mutants (RAP) or wild type (DMSO). (D) Summary of EC_50_ values for parasites with and without RAP treatment. Mean values ± SEM are shown for either 3 or 4 cloned lines; *n* = 3 for BH296 and BH267.meta, *n* = 4 for MMV007839. Statistical significance was determined using one-way ANOVA of DMSO versus RAP EC_50_ values, followed by Sidak’s multiple-comparison post-test.

We used the G107S conditional mutant line to investigate the effect of this point mutation on parasite susceptibility to our set of PfFNT inhibitors. Our conditional system allows us to probe this in an otherwise identical parasite background, which is not possible for resistance selection where other changes besides the detected point mutation may also occur under drug pressure. We established the EC_50_ of MMV007839, BH296, and BH267.meta in either dimethyl sulfoxide (DMSO)-treated wild-type or rapamycin-treated G107S mutant parasites (Fig. 6). Mutation of PfFNT led to increases in the EC_50_ from 0.148 ± 0.048 to 47.7 ± 1.06 μM for MMV007839, from 4.04 ± 1.08 to 15.01 ± 0.746 μM for BH296, and from 0.173 ± 0.078 to 38.8 ± 1.96 μM for BH267.meta.

In conclusion, the conditional PfFNT G107S mutant line exhibited the same shift in drug sensitivity as resistant parasites obtained under drug selection with sublethal dosing, clearly showing that PfFNT is the primary target of the inhibitor class.

### *In vivo* efficacy of BH267.meta.

Finally, we tested BH267.meta *in vivo* in a P. berghei-infected mouse malaria model. For comparison, we included the established antimalarials chloroquine, mefloquine, and artesunate in the assay. Efficacy was determined by a 4-day Peters’ test (20, 21) after oral administration of 30, 10, and 3 mg kg^−1 ^day^−1^ for each antimalarial for four consecutive days. At 4 × 3 mg kg^−1^ treatment with BH267.meta, parasitemia was at 0.2% after 4 days, while doses of 10 mg kg^−1^ and higher decreased the parasitemia below the limit of quantitation (LOQ) of 0.1%. This resulted in efficacies of BH267.meta of ≥99.7% at all tested doses (Table 1). Under treatment, the mice were free of symptoms or side effects elicited by the compound. The reference compounds chloroquine, mefloquine, and artesunate were comparable at doses of 4 × 30 and 4 × 10 mg kg^−1^. Artesunate yielded lower activity at 4 × 3 mg kg^−1^. The average survival after BH267.meta treatment was similar to that after artesunate treatment and ranged from 8.0 d (3 mg kg^−1^) to 13.0 d (30 mg kg^−1^) due to recurring parasitemia (Table 1).

**TABLE 1:** *In vivo* efficacy of antimalarials, including BH267.meta, against P. berghei

Compound^*a*^	Dosing regimen (mg kg^−1^ day^−1^)^*b*^								
	30			10			3		
	Activity (%)	Survival (d)	Parasite-free mice (*n*/total)	Activity (%)	Survival (d)	Parasite-free mice (*n*/total)	Activity (%)	Survival (d)	Parasite-free mice (*n*/total)
Chloroquine	99.9	21.2	-	99.9	15.5	-	99.0	11.0	-
Mefloquine	99.9	29.1	4/5	99.9	21.0	1/5	93.0	13.0	-
Artesunate	99.0	9.2	-	99.1	8.1	-	85.0	8.8	-
BH267.meta	99.8	13.0	-	99.9	10.7	-	99.7	8.0	-

a BH267.meta was dissolved (3 mg kg^−1^ dose) or suspended (all higher doses) in 70/30 Tween 80/ethanol diluted 10-fold with water. The control compounds chloroquine, artesunate, and mefloquine were dissolved or suspended in a non-solubilizing, standard suspension vehicle (0.5% [wt/vol] carboxymethylcellulose, 0.5% [vol/vol] benzyl alcohol, 0.4% [vol/vol] Tween 80, and 0.9% [wt/vol] NaCl in water).

b Once per day on 4 consecutive days (4, 24, 48, and 72 h after infection; *n* = 3; LOQ = 0.1%). Mice with <40 parasitemia reduction were euthanized on day 4 in order to prevent death that would otherwise occur on day 6.

**FIG 7:**
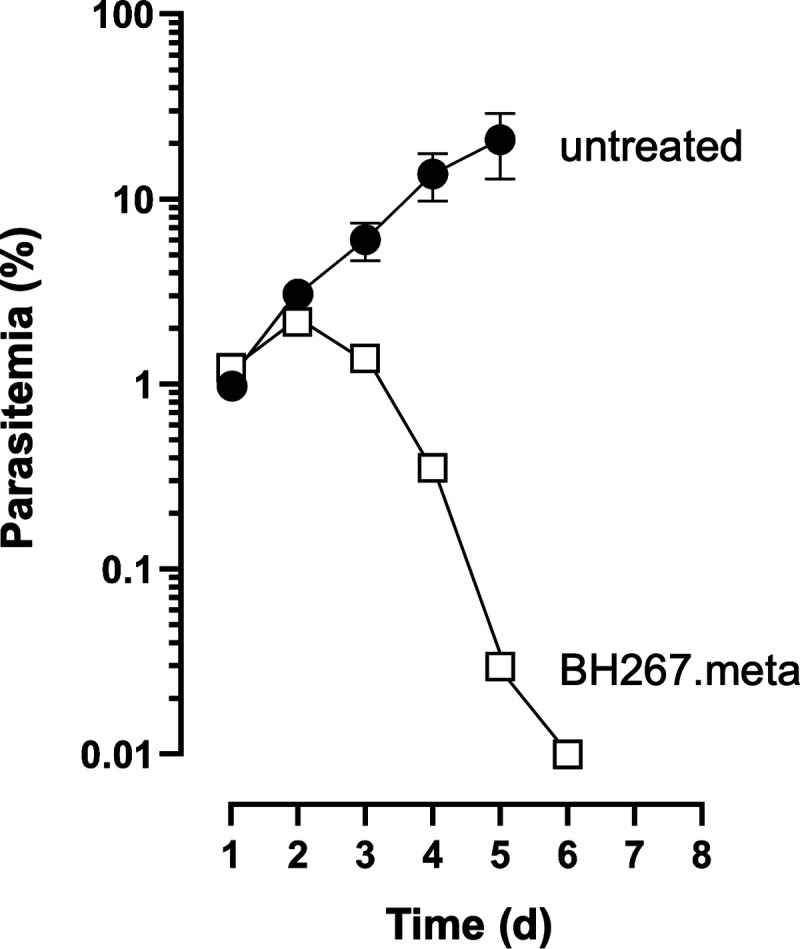
*In vivo* efficacy of BH267.meta against P. falciparum. Infected SCID mice were treated orally with four doses of 50 mg kg^−1^ on four consecutive days (squares, one animal) or left untreated as a control (filled circles, 3 animals).

In addition to the P. berghei efficacy assessment, we also evaluated BH267.meta in a P. falciparum severe combined immunodeficient (SCID) mouse model as previously described (22, 23). Efficacy was assessed following administration of 50 mg kg^−1^ d^−1^ for four consecutive days to a single animal (Fig. 7). BH267.meta decreased the parasitemia below the LOQ (0.01%), as seen previously in the P. berghei model.

## DISCUSSION

The FNT inhibitors of the pentafluoro-3-hydroxy-pent-2-en-1-one class act as analogs of the lactate substrate (12). Their molecular shape is linear (Fig. S1) with the fluoroalkyl moiety representing the protonated, neutral lactic acid transport species allowing it to enter deep into the lipophilic core of the FNT transport path (18). The deprotonated hydroxy-enone moiety represents the lactate anion species and binds farther outside toward the vestibule (17). It is conceivable that the sites of point mutations which confer resistance to the compounds are located in the transport path. The previously known PfFNT G107S mutation is located at the cytoplasmic entry site, whereas the newly found V176L position is situated deeper in the transport path (Fig. 2B). The amino acid changes at both mutation sites are rather conservative, inserting a small hydroxy-methyl (G107S) or an even smaller methylene moiety (V196L) into the original residue. As we have shown previously, the mutational flexibility of PfFNT within the transport path is very limited (12). This is related to its narrow, rigid, tube-like structure (18). Any larger residue will diminish its diameter and consequently decrease or even cease transport of the lactate substrate (8, 12), which would immediately affect the vital energy metabolism of the parasite. The PfFNT V196L mutation could possibly be overcome in a similar fashion to the G107S mutation by making the fluoroalkyl of the PfFNT inhibitors somewhat leaner to accommodate for the extra methyl in the transport path.

Previously, we identified high-affinity inhibitors by assays with yeast-expressed PfFNT and variants (12) or by direct *K_i_* measurements using fluorescence cross-correlation spectroscopy (FCCS) with solubilized protein (15) which, in some cases, poorly predict their efficacy in living parasites (17). One striking example is the compound pair BH267.meta and BH267.ortho, which differ only in the position of the pyridine nitrogen (17). Both were equally active in yeast and the FCCS assay, yet BH267.meta was more effective in parasite culture by 2 orders of magnitude (0.24 versus 14 μM). In this work, we found that the activity of BH267.meta against PfFNT G107S is more strongly affected in the parasite than in the yeast and FCCS assays. Apparently, as-yet unknown factors affect PfFNT inhibition in the parasite. For instance, it is possible that PfFNT is influenced in the parasite by the specific membrane environment, which differs in its lipid and protein composition, or that interacting proteins exist in the parasite which modulate the accessibility of PfFNT for inhibitors depending on the variant, i.e., wild-type, G107S, V196L, or G21E.

The appearance of a G21E mutation at the N terminus of PfFNT was quite unexpected since the inhibitors do not interact with this site. This mutation had some effect in culture, but only with some of the inhibitors, and the reduction in drug susceptibility was modest compared to that seen with the other two mutations. This might explain why no difference was observed with the G21E mutation in the yeast assay. The recently resolved cryo-EM structure of PfFNT shows that the N termini of the individual protomers of the homopentamer extend to the neighboring transport unit and form protein-protein interactions (18). This might foster stability in the complex and may be influenced by this mutation. However, given the limited reduction in susceptibility and specificity for only some of the inhibitors, this mutation is likely not relevant for the development of clinically relevant PfFNT inhibitors.

The key results of our study come from the parasite lines with conditional mutations in PfFNT. These data show the essentiality of PfFNT in blood-stage parasites and confirm that this protein is the primary target of the inhibitors. Previous attempts to classically knock out the *fnt* gene have failed, which already hinted at its essentiality. Now, the conditional insertion of a PfFNT transport path-blocking G107V mutation (12) leading to cell death unequivocally identifies the lactate exporter as being essential. Specific FNT inhibitors, therefore, constitute a novel principle of antimalarial action. Attacking the PfFNT lactate transporter blocks the steady-state equilibrium for the provision of glycolytic energy to the parasite (3). Concomitantly, the increased cytosolic proton concentration can be expected to interfere with the functionality of proton-coupled transmembrane transporters, among others, eliciting multi-target effects (4, 13).

Two further observations illustrate the metabolic nature of the FNT inhibitor effect: (i) gametocyte development is apparently undisturbed by a loss of lactate transport functionality, and (ii) during the blood stage, the highly metabolically active trophozoites are the ones most affected. Although we only observed gametocyte development over the course of 7 days in this study, if lactate transport was essential, we would expect to see an impact on early development. We did not perform further experiments because previous studies testing the PfFNT inhibitors during sexual development showed only a very modest reduction during stage-I parasite development, but not in later stages (24). Data on the energy metabolism of gametocytes are still scarce (25); it was found that the main glycolytic product of gametocytes is acetate-CoA, which may feed into a functional aerobic tricarboxylic acid (TCA) cycle (26, 27). Furthermore, lipid consumption is increased in gametocytes, equally leading to acetate-CoA (28). Thus, the metabolic flux in gametocytes renders FNTs dispensable for the release of lactate. During the blood stage, the highest levels of lactate waste are produced by parasites in the trophozoite and schizont stages (1, 29). Accordingly, FNT inhibitors typically showed a delay in activity by 1 day, i.e., the time that parasites in the ring stage require for conversion into trophozoites. Following the delay, the PfFNT inhibitors such as BH267.meta are fast-acting, as seen by the rapid decrease in parasitemia shown in Fig. 7. Therefore, our data provide new insights into basic questions on the functionality and relevance of a working TCA cycle in asexual blood-stage parasites and gametocytes. During the blood stage, particularly in trophozoites, metabolic energy is primarily drawn from glycolysis, producing large amounts of lactate. In contrast, the generally less metabolically active gametocytes seem to have a functional TCA cycle that prevents lactate accumulation. Although we have not tested it here, further studies on the essentiality of PfFNT in liver-stage development are warranted: PfFNT inhibitors have shown strong inhibition during this life cycle stage (24), suggesting that glycolysis plays a major role in liver-stage development. The PfFNT mutants have been generated in a genetic parasite background that enables transmission in mosquitoes and conditional deletion in liver stages (30) to enable such studies in the future.

In summary, the microbe-specific FNT-type lactate transporters of human-pathogenic malaria parasites are valid novel targets, and the potential of the current inhibitor class merits further work toward their use as antimalarials with a new mode of action.

## MATERIALS AND METHODS

### Raising resistant parasites, EC_50_ determination, and sequencing of *fnt*.

P. falciparum parasites, 3D7, were cultured in RPMI 1640 medium supplemented with 0.5% Albumax with 0+ erythrocytes (University Medical Center Hamburg-Eppendorf, Hamburg, Germany) at a 5% hematocrit at 37°C in an atmosphere containing 5% CO_2_, 1% O_2_, and 94% N_2_ under standard conditions (31). To obtain resistant parasites, 50 mL of parasite culture, starting with 5 × 10^8^ to 6 × 10^8^ parasites, were exposed to 3 × IC_50_ of either MMV007839 or BH267.meta until parasitemia was established again (around 2 weeks). For sequencing of the *fnt* gene, genomic DNA from the susceptible and resistant P. falciparum parasite lines was obtained from 5 mL of culture using the QIAamp DNA minikit (Qiagen, Hilden, Germany). The primer sequences used to sequence the entire *fnt* gene are available in Table S2. To determine the EC_50_, asynchronous parasites (0.5% to 1% starting parasitemia) were exposed to serial dilutions of the drug and grown for 2 days with one change of medium (including the appropriate concentration of drug), after which the parasitemia was determined using an LSR II FACS (BD Biosciences, Franklin Lakes, NJ) as previously described (32). The data were analyzed in GraphPad Prism to determine EC_50_ values.

### Assessing stage-specific action of BH267.meta.

Asynchronous cultures containing ~2% schizonts were purified using a 60% Percoll gradient and split into 4 tubes containing 250 mL fresh blood and culture medium, shaken at 37°C for 30 min at 800 rpm to permit newly released merozoites to invade red blood cells, and then transferred into four 5-mL culture dishes. One dish was kept as a control without drug, while the others received IC_90_ BH267.meta at either the ring (12 h after invasion), trophozoite (24 h after start of the experiment), or schizont stage (48 h after start of the experiment). The cultures were maintained with daily medium changes and replenishment of IC_90_ BH267.meta when drug treatment had already commenced for a culture. Giemsa smear and flow cytometry were performed as described previously (32), and samples were taken at regular intervals (daily or every 12 h).

### Expression of PfFNT G21E and V196L in yeast, and transport inhibition assays.

The generation of codon-optimized wild-type PfFNT and the G107S mutant in the yeast expression vector pDR196 have been described previously (3, 12). Point mutations encoding G21E or V196L were introduced using the QuikChange protocol (Stratagene), and sequenced for verification (for primers, see Table S2). For phenotypic growth assays, the PfFNT mutants were expressed in Saccharomyces cerevisiae strain W303-1A Δ *jen1*Δ*ady2* cells (MATa, *can1–100*, *ade2-loc*, *his3–11,15*, *leu2–3,–112*, *trp1–1–1*, *ura3–1*, *jen1*::*kanMX4*, *ady2*::*hphMX4*) kindly provided by M. Casal (33). Yeast cultures were grown at 29°C to an optical density at 600 nm (OD_600_) of 1 ± 0.1 in selective SD medium with adenine, histidine, leucine, tryptophan, and 2% (wt/vol) glucose and lacking uracil. The cells were collected by centrifugation, washed with water, and diluted to an OD_600_ of 0.05 in 0.17% (wt/vol) Difco yeast nitrogen base without amino acids, 0.5% (wt/vol) ammonium sulfate, 50 mM morpholineethanesulfonic acid, and 1% (wt/vol) sodium l-lactate supplemented with adenine, histidine, leucine, and tryptophan. Growth at 29°C was monitored by measuring the OD_600_ at regular intervals. For measuring lactate transport inhibition, cells grown in selective SD medium were harvested at OD_600_ = 1 ± 0.1 and resuspended in 50 mM HEPES/Tris (pH 6.8) to OD_600_ = 50. Next, 1 μL of inhibitor solution in DMSO was added to 80 μL of yeast suspension 10 to 15 min prior to the assay. Transport was initiated by the addition of 20 μL lactate solution spiked with 0.04 μCi [1-^14^C]-l-lactate (Hartmann Analytic), generating a 1-mM inward gradient. Transport was stopped after 30 s by the addition of 1 mL ice-cold water and rapid vacuum filtration via GF/C filter membranes (Whatman). The filter membranes were washed with 7 mL ice-cold water and transferred to 3 mL scintillation cocktail (Quicksafe A, Zinsser Analytic), for counting (Packard TriCarb 2900 TR, Perkin Elmer Inc., Swedesboro, NJ). IC_50_ values were calculated from sigmoidal fits to a Hill equation (GraphPad Prism) from technical triplicates each for three biological repetitions.

### *In vitro* maintenance and synchronization of parasites for conditional knockout/mutation.

Asexual blood-stage P. falciparum parasites were cultured at 37°C in complete medium consisting of RPMI 1640 supplemented with 0.5% Albumax II, 25 mM HEPES, 100 μM hypoxanthine, and 10 μg/mL gentamicin. Parasites were grown at 1 to 5% hematocrit; blood was obtained from anonymous donors acquired from the National Health Service Blood and Transplant (NHSBT) service (Colindale, London, United Kingdom). Parasites were grown in a parasite gas atmosphere (90% N_2_, 5% CO_2_, 5% O_2_). Parasite cultures were synchronized by isolating mature schizont-stage parasites on a cushion of 60% Percoll (GE Healthcare). Purified schizonts were incubated at 37°C with fresh red blood cells for 1 to 4 h in a shaking incubator to allow invasion to occur. Any remaining schizonts were removed by a second Percoll purification to leave tightly synchronized ring-stage parasites.

### Plasmids and parasite transfection.

*loxP* introns for PfFNT conditional knockout and mutation were inserted with CRISPR/Cas9, using a guide targeting between the 2nd and 3rd transmembrane domains of PfFNT (34). The rescue plasmid was constructed by PCR amplifying the 5′ homology region, *loxP* intron, re-codonized domain, *loxP* intron-GFP/2nd re-codonized domain, and 3′ homology region, then Gibson assembly was used to insert all fragments into a pMK-RQ plasmid (Integrated DNA Technologies [IDT], Coralville, IA) containing a kanamycin resistance cassette. The two re-codonized domains were ordered as gBlocks (IDT). G107S and G107V codon substitutions were inserted into the second re-codonized domain using specific 5′ primers to allow conditional complementation with mutant PfFNT. The guide RNA sequence was designed with BbsI overhangs to allow insertion into BbsI cleavage sites in a pDC2 plasmid containing Cas9 under a CAM promoter, the *tracR* RNA under a U6 promoter, and a hDHFRuFCU resistance cassette for positive selection with WR99210 and negative selection by ancotil (32). Next, 60 μg of the rescue plasmid was digested by EcoRI, which was then heat-denatured at 65°C for 30 min, and combined with 20 μg of the Cas9 plasmid. Percoll-enriched egressing schizonts were electroporated using an Amaxa P3 Nucleofector kit (Lonza) and selected after 24 h with 2.5 nM WR99210, which was added daily for 4 days. Correct integration of all transfectants was confirmed by PCR (Fig. S3). To obtain a clonal population, parasites were serially diluted in a flat-bottomed 96-well plate, and wells containing single plaques after 10 to 14 d were expanded and verified by PCR. For all experiments, ring-stage parasites were treated with either 100 nM rapamycin or DMSO for 4 h, and excision of the four C-terminal transmembrane domains was confirmed by PCR (Fig. S3).

### Determination of growth and EC_50_ of conditional PfFNT knockout/mutation lines.

Parasitemia was measured by flow cytometry from tightly synchronized parasite lines (PfFNT cKO and conditional mutants G107S and G107V; *n* = 3). Five wells, each containing 200 μL of culture, adjusted to approximately 1% parasitemia and 4% hematocrit, were treated with DMSO or rapamycin for 4 h. Every 24 h, one well from each line/condition was fixed in 2% paraformaldehyde/0.2% glutaraldehyde in phosphate-buffered saline, over 5 days in total. Parasites were stained with SYBER Green and parasitemia was measured by flow cytometry on a LSRFortessa flow cytometer (Becton, Dickinson, Franklin Lakes, NJ) using FACSDiva software. Data were analyzed using FlowJo10 analysis software (Becton, Dickinson). For EC_50_ determination of the PfFNT G107S line, dilution series of MMV007839, BH296, and BH267.meta were added to the wells in 50 μL of RPMI 1640 medium. Rapamycin- or DMSO-treated parasites at a starting parasitemia of 1% and 2% hematocrit were added to the central wells of 96-well plates, 50 μL well, with the outside wells filled with complete medium. The parasites were grown for 96 h, frozen overnight, and thawed the next day. Next, 25 μL of 5× lysis buffer was added to each well (20 mM Tris-HCl [pH 8.0], 2 mM EDTA, 1.6% Triton X-100, 0.16% saponin, 5× SYBR Green). Plates were incubated in the dark at room temperature for 2 h, and the fluorescence intensity was determined (FLUOStar Omega plate reader; BMG Labtech) with excitation and emission filters of 485 and 520 nm, respectively. The experiments were done in technical triplicates and repeated 3 to 4 times. EC_50_ values were calculated for each biological replicate using nonlinear robust fit regression (GraphPad Prism).

### *In vivo* antimalarial efficacy of BH267.meta.

*In vivo* efficacy of BH267.meta against P. berghei was determined as previously described (20, 21). Mice were infected with a GFP-transfected P. berghei ANKA strain (donated by A. P. Waters and C. J. Janse, Leiden University, Leiden, The Netherlands), and parasitemia was determined using standard flow cytometry techniques. The detection limit was 0.1%, i.e., 1 parasite in 1,000 erythrocytes. Activity was calculated as the difference between the mean parasitemia for the control and treated groups (*n* = 3, each) expressed relative to the control group. BH267.meta was dissolved (3 mg kg ^−1^ doses) or suspended (all higher doses) in 70/30 Tween 80/ethanol, diluted 10-fold with water, and administered orally as four consecutive daily doses (4, 24, 48, and 72 h after infection). Four different doses of BH267.meta were analyzed (30, 10, and 3 mg kg ^−1^). *In vivo* efficacy against P. falciparum was conducted at The Art of Discovery, Derio, Spain according to the standard assay previously described (22, 23). Animal experiments against P. berghei were carried out at the Swiss Tropical and Public Health Institute and adhered to local and national regulations of laboratory animal welfare in Switzerland (awarded permission no. 1731). Protocols are regularly reviewed and revised following approval by the local authority (Veterinäramt Basel Stadt).

### Virtual docking.

Docking was done using Maestro (version 12.8, release 2021-2, Schrödinger). The PfFNT protein structure (PDB no. 7E27) was imported for generation of a PfFNT V196L model. The amino acid exchange was processed using the Protein Preparation Wizard within Maestro. The different inhibitors were docked using the ligand-centered IFD protocol (35), in which both the ligand and the protein binding pocket were flexible. The highest IFD score, i.e., the most plausible binding pose, was chosen for display.
