# Supplementary material for: The Plasmodium Lactate/H+ Transporter PfFNT Is Essential and Druggable In Vivo
Source: Antimicrob Agents Chemother. 2023 Jul 10;67(8):e00356-23. doi: 10.1128/aac.00356-23 (PMC10433847; doi:10.1128/aac.00356-23)
Supplement: Supplemental file 1 — Supplemental material. Download aac.00356-23-s0001.pdf, PDF file, 3.0 MB [file aac.00356-23-s0001.pdf]

Table S1. Significance tests (p-values; Student's t-test, unpaired) of compound activities (MMV007839 – 7839; BH267.meta – 267.*m*; and BH296 – 296) against 3D7 parasites and selected resistant strains with PfFNT mutations G107S, G21E, and V196L. Non-significance ($p > 0.05$) is indicated by yellow cell shading, borderline significance by a hash (#).

	**3D7** 7839	**G107S** 7839	**G21E** 7839	**V196L** 7839	**3D7** 267.*m*	**G107S** 267.*m*	**G21E** 267.*m*	**V196L** 267.*m*	**3D7** 296	**G107S** 296	**G21E** 296	**V196L** 296
**3D7** 7839	—	<0.0001	0.0044	<0.0001	0.0008	<0.0001	0.0061	<0.0001	<0.0001	<0.0001	0.0001	0.0010
**G107S** 7839		—	<0.0001	<0.0001	<0.0001	**#0.0401**	<0.0001	<0.0001	<0.0001	<0.0001	<0.0001	**0.0846**
**G21E** 7839			—	<0.0001	0.0081	<0.0001	0.0058	<0.0001	0.0011	0.0001	0.0002	0.0012
**V196L** 7839				—	<0.0001	0.0001	<0.0001	**0.1370**	<0.0001	0.0024	0.0092	0.0235
**3D7** 267.*m*					—	<0.0001	0.0041	<0.0001	0.0001	<0.0001	0.0001	0.0010
**G107S** 267.*m*						—	<0.0001	0.0003	<0.0001	<0.0001	<0.0001	0.0259
**G21E** 267.*m*							—	<0.0001	0.0001	<0.0001	0.0001	0.0010
**V196L** 267.*m*								—	0.0001	0.0010	0.0047	**#0.0454**
**3D7** 296									—	0.0006	0.0006	0.0017
**G107S** 296										—	**0.3687**	0.0016
**G21E** 296											—	0.0077
**V196L** 296												—

Table S2. Primers for genomic *fnt* fragment amplification, sequencing, and point mutations

Primers for fragment amplification from gDNA

Set 1 (1111 bp):

Primer 54 forward
CGAT GCGGCCGC TAA GAGGGTTGTTTTATTATCAC

Primer 56 reverse
CTGACCTAGGACTACCGCTGCCGGACCCAGATCCTGAACCATTTCGTAATTCTATAGATAA
A

Set 2 (1889 bp):

Primer 300 forward
CCATTTGATTATTGTGATTGAAAGTG

Primer B14 reverse
ACACCTGGGATAATTAAATAATCATTTATTTTTTGAG

Primers for fragment sequencing

Primer 54 forward, seq. s. above

Primer B14 reverse, seq. s. above

Primer 186 forward
TATAGCGGCCGCTAACCACCAAATAATTCCAAATATGTTTTAGATCC

Primer B13 forward
TATCATATATTTTATCATTTATTCCATCA

Primer B12 reverse
TGTACAAAGTATAAACACTTTATGCATACAC

Primers for point mutations (mutation site in lower case, codon underlined)

Primer G21E forward
CAAAAGCGTGTGCGGAGaGGAAGAAAGCTATATC

Primer V196L forward
GGTTGTAACATATTTtTATGCTTGGCGGTGTAT

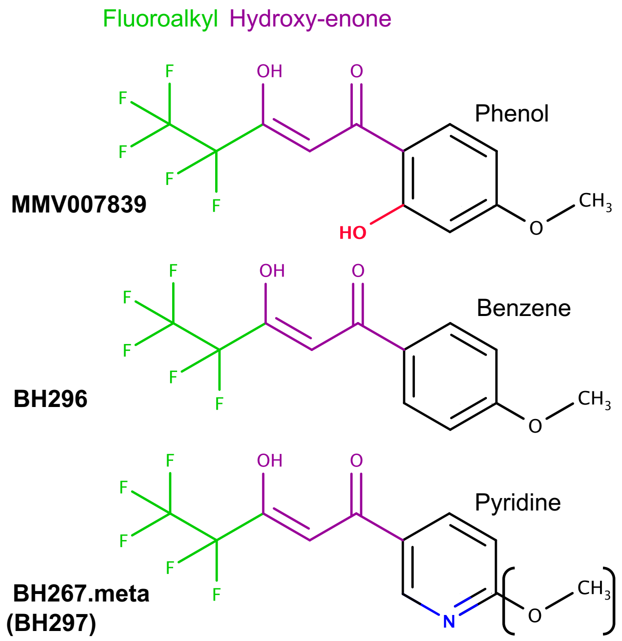

Fig. S1. Molecular structures of the PfFNT inhibitors used in this study.

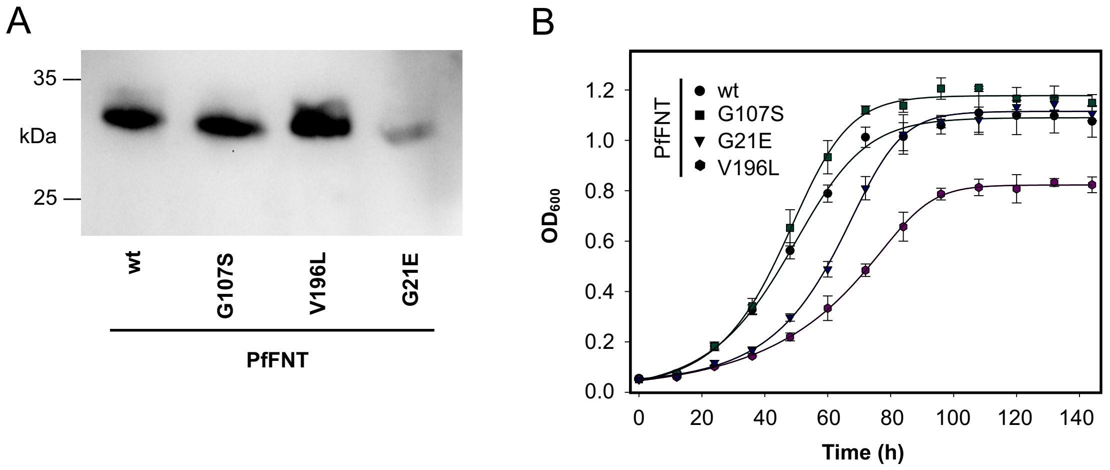

Fig. S2. Expression of wildtype and mutant PfFNT in yeast and growth on lactate media. **A.** Western blot using a primary antibody directed against an N-terminally engineered hemagglutinin epitope. **B.** Yeast lacking endogenous monocarboxylate transporters but expressing PfFNT wildtype or G107S, G21E, or V196L mutants were grown in liquid media containing lactate as the sole carbon source.

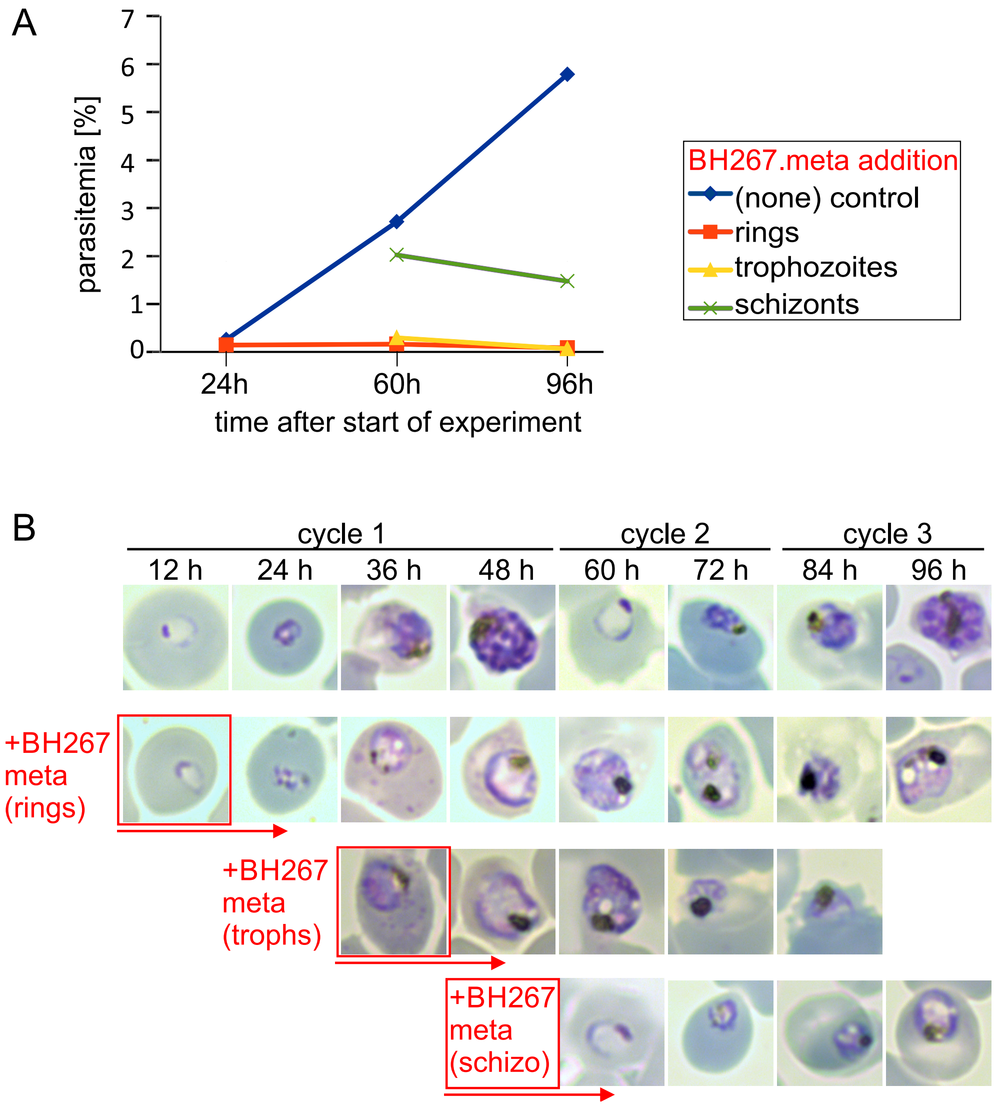

Fig. S3. Experiment similar to that shown in Fig. 3 of the main paper showing growth (A) and the parasite stage in Giemsa smears (B) in a synchronised culture split into 4 dishes consisting of a control (no drug), or cultures where BH267.meta was added at the ring stage, the trophozoite stage or the schizont stage. Parasitemia (A) and Giemsa smears (B) were taken at the indicated time points.

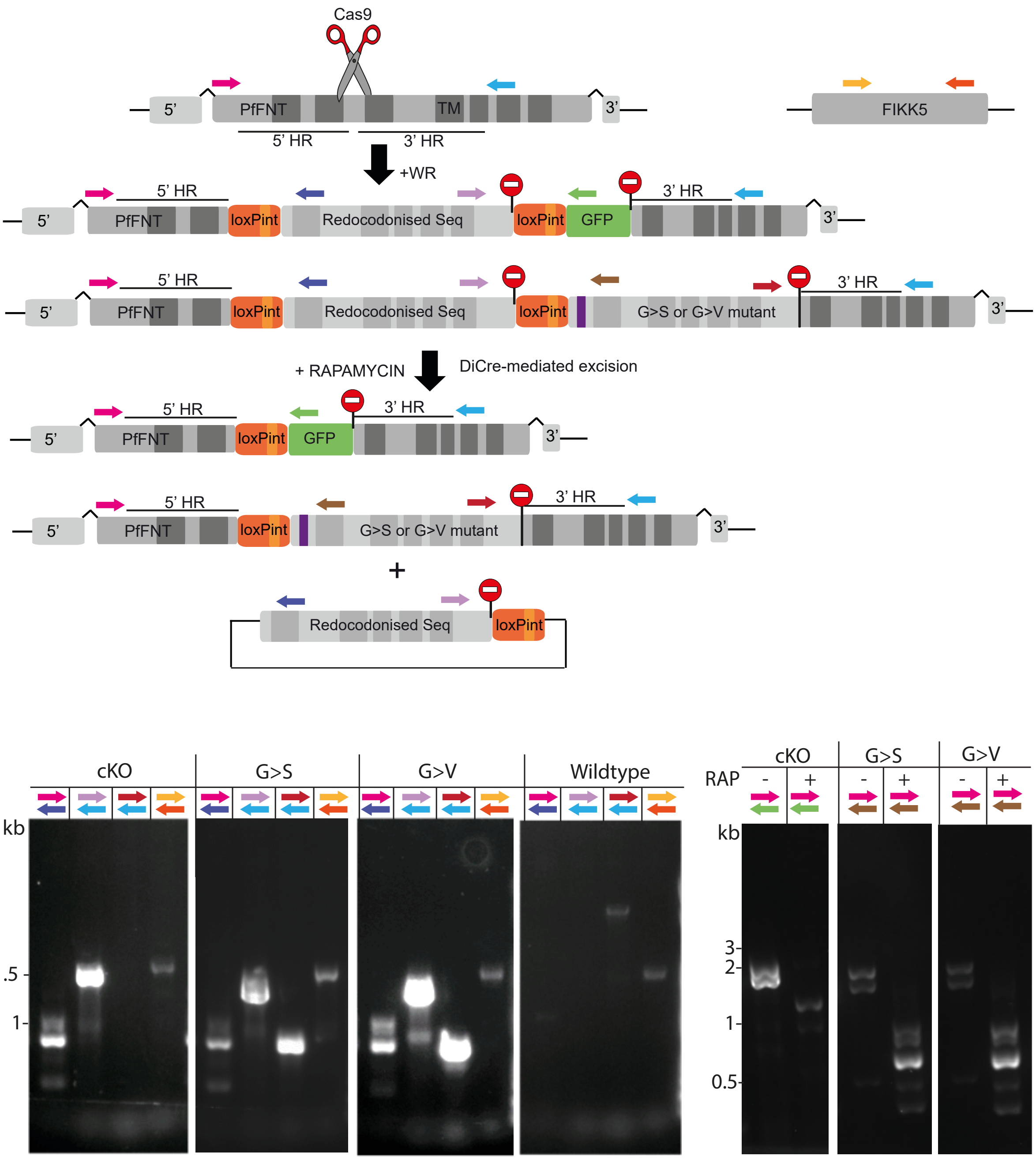

Fig. S4. Schematic of the conditional knockout and mutation construction (A) and confirmation by PCR (B). See Methods section in the main paper for details.
